# Measuring clinical outcomes of highly multiplex molecular diagnostics for respiratory infections: A systematic review and conceptual framework

**DOI:** 10.1017/ash.2022.362

**Published:** 2023-01-09

**Authors:** Tristan T. Timbrook, Tara B. Wigmosta, Rachael B. Hemmert, Jonathan B. Dimas, Alexander Krause, Sébastien Spinali, Meghan Thelen, Isabelle Tongio, Jean-Louis Tissier

**Affiliations:** 1 BioMérieux, Marcy l’Etoile, France; 2 University of Utah College of Pharmacy, Salt Lake City, Utah, United States; 3 University of Utah College of Nursing, Salt Lake City, Utah, United States

## Abstract

**Objectives:**

To review methodologies and outcomes reporting among these studies and to develop a conceptual framework of outcomes to assist in guiding studies and production of clinical metrics.

**Data sources:**

PubMed and Embase from January 1, 2012, thru December 1, 2021.

**Study eligibility criteria:**

Studies evaluating highly multiplex molecular respiratory diagnostics and their impact on either clinical or economic outcomes.

**Methods:**

A systematic literature review (SLR) of methodologies and outcomes reporting was performed. A qualitative synthesis of identified SLRs and associated primary studies was conducted to develop conceptual framework for outcomes.

**Results:**

Ultimately, 4 systemic literature reviews and their 12 associated primary studies were selected for review. Most primary studies included patient outcomes focusing on antimicrobial exposure changes such as antibiotic (80%) and antiviral use (50%) or occupancy changes such as hospital length of stay (60%). Economic outcomes were infrequently reported, and societal outcomes, such as antibiotic resistance impact, were absent from the reviewed literature. Qualitative evidence synthesis of reported outcomes yielded a conceptual framework of outcomes to include operational, patient, economic, and societal domains.

**Conclusions:**

Our review highlights the significant heterogeneity in outcomes reporting among clinical impact studies for highly multiplex molecular respiratory diagnostics. Furthermore, we developed a conceptual framework of outcomes domains that may act as a guide to improve considerations in outcomes selection and reporting when evaluating clinical impact of these tests. These improvements may be important in synthesizing the evidence for informing clinical decision making, guidelines, and financial reimbursement.

The burden of antimicrobial resistance (AMR) is a public health threat that cannot be overstated. The World Health Organization (WHO) has classified AMR as one of the top 10 global public health threats.^1^ Current reports estimate that AMR causes 1 death every 15 minutes in the United States. Mortality may reach 10 million deaths per year worldwide by 2050, exceeding the mortality rates of cancer.^2,3^ Antibiotic use is the primary driver of AMR, and inappropriate antibiotic use is well established in 50% of respiratory cases.^4^ The need for antimicrobial stewardship solutions to combat AMR is clear.

Innovative diagnostic tools combat AMR by improving stewardship programs and thus the appropriate use of antimicrobials.^5–7^ Reporting of outcomes among diagnostic studies is generally heterogenous, which is a barrier to summarizing the literature in respiratory diagnostics.^6^ Often studies focus only on performance of the assay but not consistently or comprehensively measuring downstream impact on patient management (eg, therapy changes), related health outcomes (eg, mortality), economic costs (eg, payer or patient perspective), or more broadly, societal impact through AMR and population-level economic measurements.^5,6,8^ Opportunities appear to exist in improving outcomes selection and reporting among studies that evaluate the value brought by highly multiplex respiratory diagnostic solutions.

Improved approaches toward the measurement of outcomes is important for informing use, summarizing evidence for guidelines, decisions, recommendations, and determining the entire value for reimbursement.^7^ Given the diversity in outcomes reporting among studies, a comprehensive analysis of currently reported outcomes for these innovative technologies is needed along with the application of a valuation framework to aid in the identification of gaps and facilitate future outcomes selection and reporting. Achieving this goal may be accomplished through the development of a conceptual framework or a network of interlinked concepts that provide a comprehensive understanding of a phenomenon (eg, test–treatment–outcome pathway), which may be informed by the literature.^9^

Our ultimate objective is to develop a conceptual framework for clinical and economic value outcomes for infectious diseases multiplex respiratory diagnostics. We performed an umbrella review (ie, a review of systematic reviews) to determine the current state of the literature for outcomes reporting among highly multiplex molecular respiratory diagnostics studies and to inform framework development.

## Methods

### Literature search

Systematic literature reviews were obtained from PubMed and Embase between January 1, 2012, and December 1, 2021. Studies from 2012 onward were selected due to the availability of commercial rapid multiplex testing at this time.^6^ The following search strategy was employed: [(systematic review OR meta-analysis) AND (point of care OR rapid OR bedside OR real time OR near patient) AND (test OR assay OR PCR OR molecular OR diagnostic)] and [(virus AND respiratory infection OR pneumonia OR bronchitis OR CAP OR acute respiratory illness OR ARI OR chronic obstructive pulmonary disease OR COPD OR Asthma OR Influenza-Like Illness OR ILI OR non-pneumonic lower respiratory tract infection OR LRTI OR sepsis)]. Search results were imported into a citation manager for review (EndNote 20, Thomson Reuters, New York).

### Study selection

Systematic literature review (SLR) articles were included if they evaluated a highly multiplex molecular diagnostic test that included at least 1 clinical or economic outcome. Highly multiplex diagnostic tests were defined as tests with 6 or more respiratory targets to align with cutoffs determined by the billing and coding for the Centers of Medicaid and Medicare Services. To focus on real-world evidence for patient outcomes, SLRs that only reported diagnostic test accuracy or economic modeling papers were excluded from the review. Additionally, abstracts from conference proceedings were excluded, and 2 investigators (T.B.W. and J.B.D.) screened the titles and abstracts for inclusion. Selected SLRs were then assessed by a full text review. Any discrepancies were resolved through consensus with a third author (T.T.T.). Primary studies from the selected SLRs were then reviewed for inclusion if they evaluated a highly multiplex molecular diagnostic test that included at least 1 clinical or economic outcome. The selected primary studies were then reviewed for study characteristics, quality assessments, and outcome evaluations to inform the conceptual framework of outcomes. Descriptive data were only reported for comparative effectiveness outcomes of diagnostics studies. Additionally, studies describing noncomparative effectiveness of diagnostics outcomes were utilized for conceptual framework evidence synthesis. These types of diagnostic outcomes help identify additional relevant characteristics such as effect modifiers and effect mediator variables. Data extraction was completed by 2 investigators (T.B.W. and J.B.D.). Authors were not contacted for missing data.

### Quality assessments

Quality assessments on the selected primary studies were completed by 2 investigators (T.B.W. and J.B.D.) using the Cochrane tools of ‘Risk Of Bias In Non-randomized Studies of Interventions’ (ROBINS-I) for all included observational studies and ‘Risk of Bias 2’ (ROB 2) for randomized trials.^10,11^ Differences in quality assessments between reviewers were resolved through consensus. ROBINS-I appraises bias potential in 7 domains including bias related to confounding, patient selection, classification of the interventions, deviations from intended interventions, missing data, measurement of outcomes, and selection of the reported results. ROB 2 appraises 5 domains of bias in randomized trials including bias arising from randomization process and selection of the reported result.

### Synthesis of outcomes

The conceptual framework development involved an iterative process that included identifying relevant literature and extracting relevant concepts from the chosen publications. The concepts were then categorized and mapped to fully understand the complex relationship of diagnostics and value outcomes.^9^

Each selected study by was analyzed and cross validated by 2 reviewers (T.B.W. and J.B.D.) to achieve consensus regarding key concepts and their contribution to the overall framework.^9^ Each reviewer read the selected studies to identify key concepts related to diagnostic outcomes. These concepts were categorized into a matrixed concept table. The concepts then were furthered analyzed to clarify definitions; to reduce redundancy; and to understand the roles, assumptions, and connections among the various concepts. Lastly, concepts were further organized into discrete domains that were used to develop the conceptual framework. Face-to-face meetings (in person or video conferencing) were held to build consensus and resolve discrepancies. An iterative process was used to account for negative cases, to account for ambiguity or variation, and to describe key concepts found within existing literature.

## Results

**Fig. 1.**
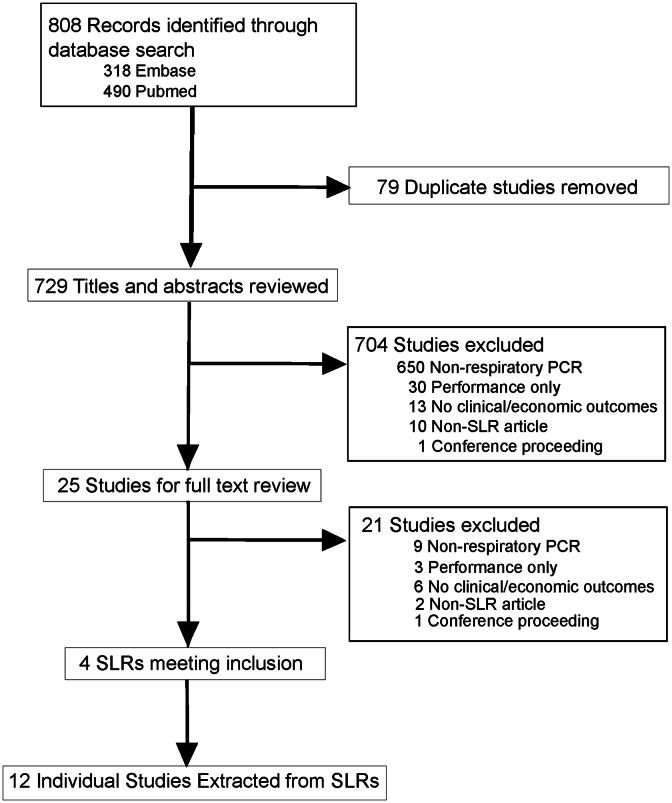
Study selection flow diagram.

The literature search criteria resulted in 808 publications meeting the search criteria; of these, 79 were duplicates (Fig. 1). In total, 704 publications were excluded by titles and abstracts review related to characteristics including nonrespiratory PCR, performance only, lack of clinical or economic outcomes, nonsystematic literature review, or conference proceedings resulting in 25 SLRs for full review. Upon full-text review of these articles, 21 additional SLRs were removed leaving 4 SLRs for inclusion.^6,12–16^ These SLRs were reviewed for the primary studies with highly multiplex molecular diagnostics that produced 12 primary studies of 7,639 patients for analysis.^17–28^

Characteristics of the included primary studies are shown in Table 1. Most primary studies were cohort studies without a control group. Randomized controlled trials comprised the minority of primary studies (33%). More primary studies included adult participants than pediatric patients (58% and 33%), and only 2 studies included both adult and pediatric populations. Eligibility criteria varied greatly with patients who had a confirmed positive respiratory test as the most common enrollment. In contrast, test setting was nearly uniform; inpatient and/or emergency departments was the most common setting. Respiratory diagnostic cointerventions (eg, biomarkers, antimicrobial stewardship prospective audit and feedback, local clinical guidelines, etc) were not commonly reported. Finally, using the ROB 2 and ROBINS-I quality assessments (Supplementary Tables S1 and S2), most primary studies (90%) had at least a moderate risk of bias, often related to confounding among observational studies.

**Table 1.** Primary Study Characteristics

Study Characteristics	No. (%)
**Study design**	
Randomized controlled trial	4 (33)
Quasi-experimental	3 (25)
Cohort study without a control group	5 (42)
Cohort study with a control group	0 (0)
**Setting**	
Inpatient	6 (50)
Outpatient	0 (0)
Both	6 (50)
Inpatient and ED encounters	4 (66.7)
Inpatient, ED, and clinic encounters	2 (33.3)
**Patient population**	
Pediatric	4 (33)
Adult	7 (58)
All	1 (8)
Not reported	0 (0)
**Eligible patients**	
ILI	3 (25)
URTI	1 (8)
Other	11 (92)
**Cointerventions**	
Biomarkers^a^	3 (25)
ASP	1 (8)
Local guidelines	0 (0)
Other	0 (0)

Note. ED, emergency department; ILI, influenza-like illness; URTI, upper respiratory tract infection; ASP, antimicrobial stewardship program.

a Biomarkers included 3 procalcitonin studies as cointerventions. An additional 2 studies had C-reactive protein without being a specific intervention.

**Fig. 2.**
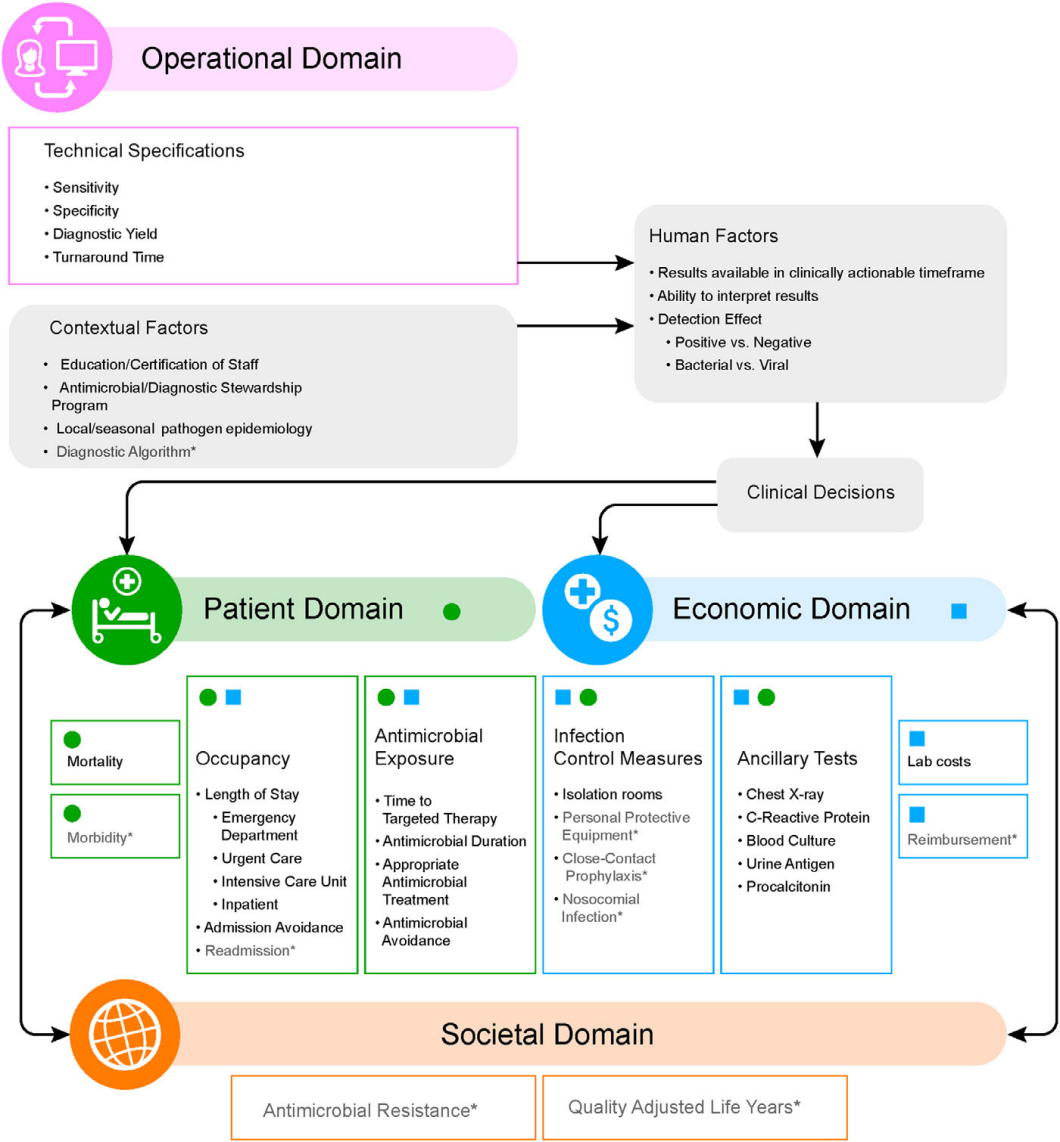
Conceptual framework of outcome measures.

The conceptual framework of the impact of highly multiplex molecular diagnostic testing affecting clinical outcomes is shown in Figure 2. From the evidence synthesis (Supplementary Table S3, concept matrix), 4 overall overarching domains emerged from the outcomes reported in the literature: operational, patient, economic, and societal. A description of domains and summary of examples are provided in Supplementary Table S4 (online). The operation domain encompasses the technical specifications of diagnostics tools (eg, sensitivity, specificity, turnaround time, etc) as well as operational resources and contextual factors at study sites (eg, education-level of staff, presence or absence of antimicrobial stewardship programs, local/seasonal pathogen epidemiology). These operational factors influence a clinician’s ability to receive and interpret results, which in turn affects the clinical decisions made in the diagnosis and treatment of patients. These decisions have both direct and indirect influences on the remaining 3 interconnected domains: patient (eg, mortality, length of stay, antimicrobial exposure), economic (eg, laboratory costs, ancillary testing, the use of isolation rooms), and societal (eg, antimicrobial resistance).

**Fig. 3.**
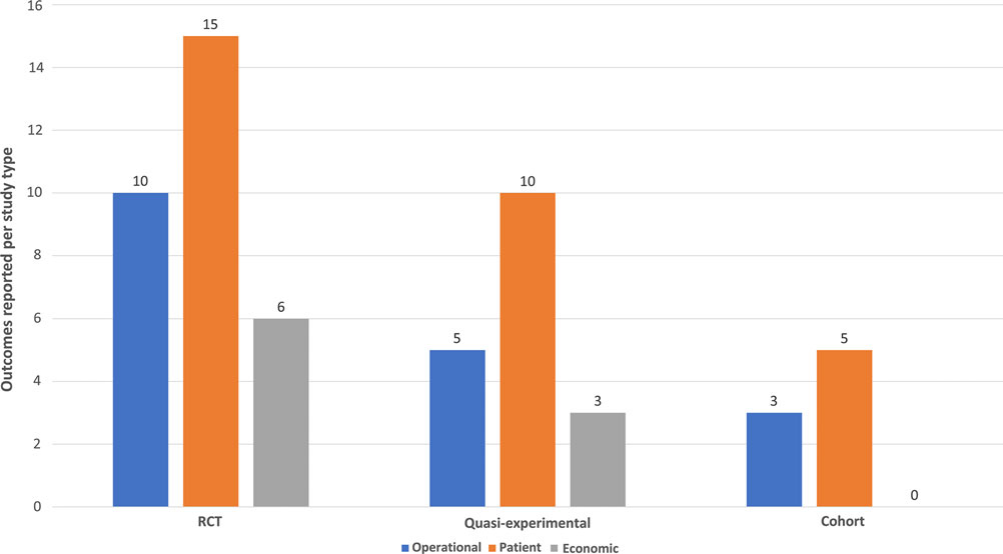
Frequency of outcomes reported per study type. *Frequencies based on presence of domain metrics (eg, length of stay) within individual studies.

Clinical outcomes reported for primary studies were quite variable (Fig. 3). In the context of the conceptual framework, outcomes were more adequately reported in randomized controlled trials. In contrast, implementation and cohort studies reported limited outcomes. Economic measures were infrequent regardless of study type. Societal outcomes (eg, AMR) were absent in the reviewed literature.

Although our study focuses on clinical outcomes in the postanalytical phase, most (80%) of the included studies notably reported operational domain elements, specifically analytical turnaround time, as an outcome. Outcomes from this category also included diagnostic yield (100%) and performance (20%). Overall, outcomes within the patient domain were well represented. The duration of therapy was reported in 90% of studies, and antibiotic and antiviral prescriptions were reported at 80% and 50%, respectively. Although the length of stay (LOS) was reported in 60% of studies reviewed, only 30% reported hospital admission rates. Mortality outcomes were reported in 40% of studies. With respect to the economic domain, the use of isolation rooms was most commonly reported (20%), followed by use in ancillary testing (20%) and laboratory costs (20%). Notably, the operational definitions of outcomes were often heterogenous when reported (eg, LOS defined by total hospital stay vs time from culture).

## Discussion

In our current review, we report on 4 systematic reviews with 12 primary highly multiplex molecular respiratory diagnostic studies evaluating clinical impact. Although operational and patient outcomes were consistently reported, economic outcomes were rarely reported and societal outcomes never reported. Our review highlights the heterogeneity in outcomes selection and reporting for studies evaluating the clinical impact of highly multiplex molecular respiratory diagnostics. Furthermore, our conceptual framework of outcomes (operational, patient, economic, and societal domains) provides a basis as a planning tool to improve the selection and reporting strategy toward the design, analysis, and communication of evidence in evaluations of highly multiplex molecular respiratory diagnostics. We believe that a more standardized reporting of outcomes is an opportunity to more precisely measure population-level effects of diagnostics (eg, AMR). The potential of measuring the population-level impact of diagnostics and their public health impact was recently described by the ASM Clinical and Public Health Microbiology Committee and ASM Corporate Council.^29^ They noted that these benefits (ie, improvements of antibiotic use and decreases of community-acquired resistance) would be possible through collaborative ventures and robust implementation strategies (ie, antimicrobial stewardship and operational strategy). Current single-center studies are perhaps unlikely to report AMR because these effects are small and such studies are underpowered to measure the outcome.

Our conceptual model has several implications. Application of conceptual framework may facilitate greater consistency in conduct and reporting of outcomes to inform both researchers and clinicians. The framework communicates the intricacy of connected factors along the test–treatment–outcome pathway in the patient encounter.^8,30^ For clinicians, it elucidates where to target metric recording for internal reporting by laboratories and antimicrobial stewardship teams to internal stakeholders. This method may help support the sustained use of the diagnostic along with identification of opportunities for improved use within individual institutions. Moreover, many of these metrics in the conceptual framework are routinely measured by ASPs (eg, occupancy, antimicrobial exposure, and morbidity and mortality), which should facilitate streamlined assessments of diagnostics using the metrics. However, some measures (eg, technical specifications, infection control measures, ancillary tests, laboratory costs) may require collaborations with infection prevention and control in addition to microbiology to enable sharing of data for comprehensive metric consolidation.^31^ Similarly, for researchers, the framework demonstrates the cascading outcomes and importance of standardizing measurement along the pathway for consistent communication of value. Beyond clinicians and researchers, improved selection and reporting of outcomes may enable health authorities and private payers to better assess the value of diagnostics. Finally, the framework may have potential in generalizing or adaptation to other infectious diseases diagnostics and related syndromes.

The use of the conceptual model gives considerations on important domains of outcome selection and reporting. In our review of the literature, clinical management changes were most often measured. This is intuitive given the hierarchical efficacy of the test–treatment–outcome pathway (ie, testing accuracy and timeliness increases treatment effectiveness which increases clinical outcome effectiveness). Changes in clinical management are the most proximal and easily measured impact of diagnostics.^8^ However, changes in decision making and differential diagnoses are more proximal to the diagnostic result impact yet are resource intensive and logistically challenging to measure. Although pre- and postanalytical implementation strategies were reported in our review, they were very infrequent. These cointerventions, such as education of staff and diagnostic algorithms (eg, clinical decision support systems and biomarkers such as procalcitonin), have demonstrated favorable impacts on the clinical effectiveness of highly multiplex molecular respiratory diagnostics on patient outcomes. For other molecular diagnostics, cointerventions such as antimicrobial stewardship, have proven essential.^5,32,33^ Greater consistency in selection and reporting of pre- and postanalytical implementation strategies, along with increased use thereof, has significant potential to improve both the effectiveness of highly multiplex molecular respiratory diagnostics within institutions and the understanding and breadth of evidence in the literature.^34^ When using the conceptual framework, researchers must be conscientious in the selection of individual outcomes. For example, with the patient outcome domain, in some countries the proportion and time to neuraminidase inhibitor use for influenza patients may be an appropriate outcome to be measured, and in other countries, neuraminidase inhibitor use is significantly limited.^35^ Therefore, local clinical practice, standards, and resource limitations must be kept in mind when selecting certain individual outcomes. Conversely, efforts to decrease antibiotic use in viral respiratory presentations are nearly consistent across settings to and thus are universally applicable. Although individual outcomes may not be applicable across all settings, the overall outcome domain categories presented are likely generalizable from this predominantly inpatient literature to decentralized settings.

Notably, the quality of most studies suggested at least a moderate risk of bias within our SLR. In fact, we only noted 4 randomized controlled trials, which serve as the gold standard in evidence-based medicine clinical outcomes studies due to their unconfounded results. Additionally, in examining the quality assessments, only a small minority (17%) of real-world evidence studies adjusted for confounding when evaluating outcomes, thus leaving the majority subject to potential bias in estimates of effective that could be due to imbalances in baseline patient characteristics. Beyond the production of more randomized controlled trials, real-world evidence studies would greatly benefit in adjusting for confounding with causal modeling to reduce potential for these biases.^36^ Similarly, sample size justifications were rarely reported; therefore, most studies were unclearly powered. Generally, clinical assessment trials on outcomes of diagnostic tests need to be larger than trials of therapeutic treatments; thus, it is strongly desirable for future studies to provide these sample size justifications.^30^ Finally, opportunities were present in the selection and reporting of operational definitions of outcomes. For instance, the measure of time-dependent outcomes (eg, antibiotic use, LOS, etc) were often derived for the total patient encounter whereas the diagnostic test can only impact post result outcomes and thus biased estimates of effect are often reported.^37–39^

This study had several limitations. Our review’s main focus was on the framework for outcomes to ensure the effectiveness of highly multiplex molecular respiratory diagnostics is improved in selection and reporting of outcomes, yet preanalytical factors (eg, timing of test in management of encounter) and postanalytical factors (eg, adjustment to microbiology results presentation and communication) are known to impact the clinical efficacy of diagnostics.^30,34,40^ However, we believe that greater consistency in selection and reporting of study clinical outcomes are vital while pre- and postanalytical implementation strategies, though essential to report, may be more heterogenous based on local needs. Broad conceptual framework mappings of the full test–treatment–outcome pathway have been described elsewhere.^30,41^ Similarly, our data are predominantly from the inpatient setting and emergency departments. As respiratory diagnostic testing moves to the outpatient setting of urgent-care clinics, medical offices, and retail clinics, reassessment should be performed because information needed to inform outcome selection is limited.^42^ Finally, safety outcomes were lacking in the reviewed literature and were not evaluated for the framework. These outcomes may be particularly important as highly multiplex molecular respiratory diagnostics expand into outpatient settings as substantial opportunity in prescription avoidance exists.

In conclusion, based on the current literature, there is significant heterogeneity in the selection and reporting of general domains and individual clinical outcomes in the test–treatment–outcome pathway. We developed a conceptual framework of outcomes to improve future research studies and clinical metric evaluations including operational, patient, economic, and societal domains. The use of the conceptual framework may facilitate improvements in the state of the literature for molecular respiratory diagnostics and may improve their use as well as clinical guideline development and financial reimbursement.

## Supplementary material

For supplementary material accompanying this paper visit https://doi.org/10.1017/ash.2022.362. click here to view supplementary material
