# Supplementary material for: Measuring clinical outcomes of highly multiplex molecular diagnostics for respiratory infections: A systematic review and conceptual framework
Source: Antimicrob Steward Healthc Epidemiol. 2023 Jan 9;3(1):e9. doi: 10.1017/ash.2022.362 (PMC9879901; doi:10.1017/ash.2022.362)
Supplement: Supplementary file 1 [file S2732494X2200362Xsup001.docx]

**Supplementary** **Appendix**

**Supplementary** **Table 1.** ROBINS-I Quality Assessments

| Author | Baseline confounding | Selection of participants |  | Classification of intervention | Deviation from intended intervention | Missing data | Measurement of outcomes | Selection of reported results |  | Overall risk of bias |
| --- | --- | --- | --- | --- | --- | --- | --- | --- | --- | --- |
| Rogers  (2015) | Low | Moderate |  | Moderate | Low | Moderate | Low | Moderate |  | Moderate |
| Rappo  (2016) | Moderate | High |  | Low | Low | Moderate | Moderate | Low |  | High |
| Keske  (2018) | High | Low |  | Low | Low | Moderate | Moderate | Low |  | High |
| Xu  (2013) | Moderate | Low |  | Low | Low | Moderate | Moderate | Low |  | Moderate |
| Timbrook  (2015) | Moderate | Moderate |  | Moderate | Low | Moderate | Moderate | Low |  | Moderate |
| Pettit  (2015) | High | Low |  | Low | Low | Moderate | Moderate | Low |  | High |

**Supplementary Table 2.** Risk of Bias 2 Quality Assessments

| Author | Domain A |  | Domain B | Domain C | Domain D | Domain E | Overall risk of bias |
| --- | --- | --- | --- | --- | --- | --- | --- |
| Brendish  (2017) | Low |  | Low | Low | Low | Low | Low |
| Andrews  (2017) | High |  | Low | Low | Low | Low | High |
| Gilbert  (2016) | Moderate |  | Moderate | Moderate | Moderate | Low | Moderate |
| Gelfer  (2015) | High |  | Moderate | Moderate | Moderate | Low | High |

Domain: A) bias arising from the randomization process, B) bias owing to deviations from intended interventions, C) bias owing to missing outcome data, D) bias in measurement of the outcome, E) bias in selection of the reported result. Overall risk of bias evaluated across the five domains.

**Supplementary** **Table 3.** Concept Matrix (the pre-concept map/model phase)

| **Concept Name** | **Description** | **Roles, Assumptions, Variations** | **Reported Outcomes** |
| --- | --- | --- | --- |
| Mortality | Rates of death within a given timeframe, or attributable to a specific cause. | While there are many ways mortality can be defined and operationalized, mortality metrics for diagnostic studies (when measured) frequently report either inpatient mortality or 30-day mortality. | - 30-day mortality 3% vs 5% (p=0.15) (Brendish, 2017) - 30-day mortality 4% vs 4% (p=0.79) (Andrews, 2017) |
| Clinically actionable timeframe | Availability of results within a timeframe in which clinicians can make directed decisions based upon results (as opposed to empiric treatment decisions). | Many factors contribute to results being available in a clinically actionable timeframe, such as location of testing, hours of operation, mechanisms for reporting results, speed/throughput of instruments, etc. | - Included tests if results available within 3 hours. (Vos, 2019) - Non-significant difference in antibiotic duration attributed to patients receiving antibiotics prior to randomization and/or prior to results being available to clinicians. (Brendish, 2017) - Infectious disease diagnostic results are frequently posted too late to influence clinical decisions. (Andrews, 2017) - PCR results received in ED before admission, was 13.4% (49/365) pre-RRP, and 51.5% (398/771) post-RRP. This study included only patients that were admitted to the hospital, therefore metrics on admission avoidance were absent. (Rogers, 2015) - When results were available after 6 hours, the decrease in antibiotic duration was no longer present, indicating that rapid tests had a measurable impact on physicians’ actions. (Rogers, 2015) |
| Turnaround time/throughput | Time it takes to receive test results  The amount/volume of samples that can be run on a specific instrument. | Typically compared to standard/conventional testing techniques.  Heterogenous definitions with varying start/stop points.  Full concept includes pre-analytical, analytical, post-analytical, and each study determines that start/stop points for TAT, and pre/post processes can add significant delays if not efficient.  Instruments vary widely, and resource limited settings may not have the ability to keep up with sample volumes.  Many POC instruments are low-throughput by design, as high-throughput instruments require more training and/or certified/trained staff. | - Studies unanimously found rapid molecular tests had reduced turnaround time. (Vos, 2019) - Median time to result from admission was 39.5h with standard of care, and 19h with rapid-PCR, p<0.001. (Andrews, 2017) - The mean TAT (n=230) for Luminex xTAG RVP was 46.4 h, vs. 3.1h for FilmArray (n=872), p<0.001. (Pettit, 2015) - Median TAT went from 7.7 h (rapid antigen group) to 1.7 hours (Filmarray group) for those with influenza (p=0.015). For non-influenza viruses, TAT went from 13.5 h to 1.5 hours, p<0.001. (Rappo, 2016) - Subgroup analysis of discordant results (between RAT and PCR) showed an even bigger increase in TAT (13.3h vs. 1.7h, p<0.0001). (Rappo, 2016) - Pre-RRP TAT was 1119 min (250-3705m), and 383m (72-3143m) post- RRP, p<0.001. (Rogers, 2015) - Mean rapid molecular TAT 2.3 hours [SD 1.4] vs conventional test’s 37.1 [21.5] (p<0.001) (Brendish, 2017) - Median 19 hours [IQR 8.1–31.7] vs 39.5 [25.4–57.6] (p<0.001) (Andrews, 2017) - Mean 2.1 hours [SD 0.7] vs 26.5 [15] (p<0.001) (Gilbert, 2016) - Mean 1.8 hours [SD 0.3] vs 26.7 [16] (p<0.001) (Gelfer, 2015) - Mean 6.4 hours [SD 4.9] vs 18.7 [8.2] (p<0.001) (Rogers, 2014) |
| Education/  training | Information provided to clinicians regarding the appropriate use of diagnostic tests to guide treatment decisions. | May take on many different forms/formats.  May involve different stakeholders across institutions.  May be a one-time or ongoing intervention, including the development of dedicated teams (i.e. Antimicrobial Stewardship Programs) | - Relatively low rates of antibiotic discontinuation, even when multiplex PCR and procalcitonin values indicate viral infection, indicate the need for education and/or antimicrobial stewardship interventions to guide the appropriate use/avoidance of antibiotics for respiratory infection. (Timbrook, 2015) - High rates of inappropriate antibiotic use, despite the availability of molecular respiratory tests, led to implementation of improved training sessions. There was a decrease in inappropriate antibiotic use among inpatients. Training intervention is not described. (Keske, 2018) - Training efforts may affect uptake of tests, appropriate patient selection, appropriate interpretation, and appropriate subsequent interventions. (Vos, 2019) |
| Ancillary testing | Diagnostic screening in addition to multiplex PCR, used to help guide diagnosis and/or treatment decisions. | Detection of secondary biomarkers may impact a variety of outcomes, including decision to admit/board/discharge, decision to prescribe/discontinue antimicrobials, etc. They may also provide prognostic information.  Some ancillary testing is deemed necessary (e.g. culture/susceptibility) while others may be avoided, if the “primary” test is reliable and provides the required information to guide diagnosis and treatment decisions.  Diagnostic screening tools and recommendations vary significantly, and may use laboratory, imaging, or other techniques. | **Procalcitonin (PCT)** |
|  |  |  | - Procalcitonin in combination with molecular diagnostics may further discriminate between viral/bacterial causes. (Vos, 2019) - PCT concentrations are significantly lower in those with a virus-only detection vs. those with a bacterial, or a combination bacterial-viral detection, p<0.003. (Gilbert, 2016). - PCT values reliably distinguish between viral and bacterial infection within 2-8 hours of admission, which impacts the length of empiric respiratory isolation and can guide decisions to continue antibiotic treatment before a second dose of empiric antibiotics is given. Potential reduction in costs and additional X-rays. (Gilbert, 2016) - PCT levels ≤0.1 ng/mL indicate the absence of invasive bacterial disease and support an interpretation of bacterial colonization when S. pneumoniae, H. influenzae, S. aureus, or other bacterial pathogens are detected. (Gilbert, 2016) - In 25 patients (13 standard and 12 FilmArray), the NPPCR detected a pathogenic virus without a concomitant bacterial pathogen, a clinical presentation consistent with a viral pneumonia, and a serum PCT level ≤0.1 ng/ml. Nonetheless, discontinuation of the empiric antibiotics within 48 hours of test results occurred in only 8 of the 25 (32%) patients. Despite the faster turn-around time for FilmArray patients, discontinuation of empiric antibiotics was almost identical (5 FilmArray patients and 3 standard patients). (Gilbert, 2016) - Bacterial diagnoses were made among 9%–10% of patients, with most made on the basis of sputum culture results and 25% associated with high PCT levels (>0.24 ng/mL). Three patients had positive results of a FilmArray assay for atypical bacteria, and illnesses in all 3 were associated with low serum PCT levels (Andrews, 2017) - Of the 307 patients prescribed antibiotics within the first 72 h, antibiotics were discontinued within 48 h of laboratory results in 60 (19.5%) patients with either a PCT\0.25 lg/mL, a positive PCR, or both. The median duration of antibiotics was 3 days for all of these groups and ranged from 1 to 19 days, 1 to 27 days, and 1 to 13 days for a PCT\0.25 lg/ mL, a positive RP, or both, respectively (Timbrook, 2015) |
|  |  |  | **Chest radiograph (CXR)** |
|  |  |  | - Reduction of CXR shown in a majority of high quality studies (Vos, 2019) - Multivariate log. regression (adjusting for age, immunosuppression, asthma, and ICU admission) demonstrated influenza positive FilmArray patients had a significantly lower number of CXR, p=0.005. (Rappo, 2016) |
|  |  |  | **Blood Culture** |
|  |  |  | - Blood cultures were only significant for 1 of 127 samples, indicating they are likely unnecessary in all but the patients with high pneumonia severity index (PSI) scores. (Gilbert, 2016) |
|  |  |  | **C-reactive Protein (CRP)** |
|  |  |  | - Patients with mixed infections had higher CRP values. (Laundy, 2003) |
|  |  |  | **Urine Antigens** |
|  |  |  | - Urine antigen testing for S. pneumoniae and L. pneumophila, serogroup 1 may be redundant when using multiplex PCR or NGS technologies. (Gilbert, 2016) |
| Length of stay | Time spent in the healthcare setting, typically from admission to discharge | May be defined differently in urgent care/emergency department settings. | - LOS reduction shown in a majority of high quality studies (Vos, 2019) - Mean LOS shorter in rapid molecular group (5.7d, SD 6.3) than with conventional testing (6.8d, SD7.7), p=0.0443. (Brendish, 2017) - Multivariate log. regression (adjusting for age, immunosuppression, asthma, and ICU admission) demonstrated influenza positive FilmArray patients had a significantly lower LOS, p=0.04. (Rappo,2016) - There was no difference in inpatient LOS between pre-RRP (3.4d) and post-RRP (3.2d), p-0.16. There was an increase in mean ED LOS after implementing RRP (256m pre, and 282m post, p=0.002). For viral positive patients, inpatient LOS was shorter post-RRP (3.5d pre and 3.2d post, p=0.03). (Rogers, 2015) - 55.6% (5/9) studies found significant reductions in ED LOS associated with POC testing. Mixed between antigen-based and molecular based methods (1/9 used molecular test). Two studies found no significant differences in the ED LOS of POCT and standard testing groups,18,41 whilst two studies reported that POCT increased ED LOS. (Egilmezer, 2018) - Children with influenza A CAP tended to have longer durations of fever (P = 0.20), hospital stay (P = 0.24) and total illness (P = 0.15) than those with RSV CAP, but these differences were not statistically significant (Laundy, 2003) |
| Antimicrobial Exposure | The amount of time a patient is on antibiotics, antifungals, or antivirals.  De-escalation is the removal of unnecessary antimicrobials.  Ability of test result to prevent unnecessary antibiotic use.  Ability of the test to more rapidly initiate targeted/appropriate antimicrobial therapy. | May vary based upon patient population, clinical presentation, CXR, TAT.  Significant heterogeneity between what is actually measured, including when to include multiple antimicrobials and what the start and endpoints of antimicrobial therapy are.  Many patients are treated empirically prior to test results being available, but some rapid tests allow for antimicrobials to be avoided all together.  Subgroups:  Appropriate antiviral use.  (time to start when needed, and time to stop when not needed)  Antiviral avoidance.  Antibiotic duration  Antibiotic avoidance.  Time to targeted therapy. | **Appropriate Antiviral Use** |
|  |  |  | - Improvement in appropriate antiviral prescriptions for influenza positive patients shown in a majority of high quality studies (Vos, 2019) - Appropriate neuraminidase inhibitor treatment of influenza positive patients more common in rapid molecular group (91% [52/57]) than with conventional testing (65% [24/37]), p=0.003. (Brendish, 2017) - Influenza positive patients prescribed antibiotics more often in rapid-PCR group (80%, 24/30), than with conventional testing (62%, 13/21). The time to the first dose from the time of admission was known for all but one patient in each arm and was considerably reduced in the intervention arm: median of 60.4 h in the control arm (IQR 22.7–85.2) and 24 h in the intervention arm (IQR 11.6–33.0). (Andrews, 2017) - 86% (12/14) reported increased antiviral use associated with a positive diagnosis by POCT (mix of molecular and antigen-based techniques – only one multiplex PCR). (Egilmezer, 2018) - No difference between intervention and control arm, 61% vs 61% (p=0.96). Subgroup analysis of discordant results (between RAT and PCR), showed discordant results had lower incidence of antiviral treatment (55% vs. 74%, p=0.034), and increased time to antiviral initiation (15.9h vs. 5.2h, p=0.013). (Rappo, 2016) - Mean time to d/c empiric oseltamivir amongst negative influenza patients was 4 days for Luminex xTAG RVP (n=42) and 2 days for Filmarray (n=75), p<0.001. (Pettit, 2015) |
|  |  |  | **Antibiotic Duration** |
|  |  |  | - Overall, non-significant difference in duration between rapid molecular test and conventional testing (7.2d vs. 7.7d, p=0.17), however, the proportion of single dose antibiotics was higher for the molecular group than conventional testing (10% vs. 3%, p=0.001), as was the proportion of patients with short duration (<48h) antibiotics (17% vs. 9%, p=0.0047). Post-hoc subgroup analysis looking only at those who were not prescribed antibiotics prior to randomization/result availability showed a reduction in antibiotic prescriptions for the rapid molecular group (51%, 61/120), than in the conventional testing group (65%, 107/167), p=0.0289; number needed to test to prevent one patient being treated with antibiotics is eight. An additional subgroup analysis of those with exacerbation of airway disease (as opposed to pneumonia, which requires a longer antibiotic course), showed a shorter duration of antibiotics in the rapid molecular group than with conventional testing (5.3d vs. 7.1d, p=0.0008). (Brendish, 2017) - Days of therapy per 1000 patient days (DOT, if a patient is on 3 antimicrobials on any given day, it counts as 3 DOT), was significantly lower in FilmArray group. 1560 (±895) for FilmArray vs. 2232 (±2574) for standard, p=0.03. (Gilbert, 2016) - Univariate analysis showed non-significant difference (median 23.7h vs 2 48.1h, p=0.24), however, multivariate log. regression (adjusting for age, immunosuppression, asthma, and ICU admission) demonstrated influenza positive FilmArray patients had a significantly lower duration of antimicrobial use, p=0.032. (Rappo,2016) - Overall antibiotic use (measured in days) went from 3.2d to 2.8 days after implementing RRP, p=0.003. Subgroup analysis of virus positive patients also showed a decrease from 3.2 days to 2.7d post RRP, p<0.001. (Rogers, 2015) - Mean duration of inappropriate inpatient antibiotics decreased from 9.7d to 6.2d in adult, p=0.007. In children, decreased from 6.5d to 4.6d, p<0.001. (Keske, 2018) - Implementation of rapid molecular tests did not decrease the number of antibiotic prescriptions or the duration of antibiotic treatment. (Vos, 2019) - Median 6 days [IQR 4–7] vs 6 [5–7.3] (p=0.23). (Andrews, 2017) - Mean 683/1000 patient-days [SD 317] vs 917/1000 [220], p=0.052. (Gelfer, 2015)   **Explanations/Recommendations**   - Rapid-PCR results delayed by enrollment procedures and the majority of samples being run by investigator staff, rather than clinical team. Authors propose that antibiotic decisions may have been improved if results were available sooner. (Andrews, 2017) - Because antibiotics may be prescribed prior to rapid-PCR results are available, it may be beneficial to measure the proportion of patients who are on brief-courses of antibiotics. (Egilmezer, 2018) |
|  |  |  | **Appropriate Antibiotic Use** |
|  |  |  | - Proportion of patients prescribed inpatient antibiotics was 75% using multiplex PCR and 77% using conventional testing, p=0.99. (Andrews, 2017) - Proportion of patients prescribed antibiotics pre-RRP was 73.4%, and 72.0% and RRP implementation, p=0.61. (Rogers, 2015) - Antibiotic prescription rates overall were 56.3% and 55.5% pre and post-PCR tests, respectively, p=0.92. Among hospitalized patients, antibiotics were continued inappropriately in 45% (160/359) of all inpatients, despite detection of the virus, but there was a reduction in 2016 when compared with 2015 [51.3% (81/158) in 2015 and 39.3% (79/201) in 2016, p = 0.024]. In children, the antibiotic continuation rate was 44.5% in 2015 and decreased to 28.8% in 2016 (p = 0.009); in adults, there was a decrease towards 2016 but this was not significant. (Rappo, 2016) - Twenty‐six studies assessed the effect of POCT on antibiotic prescription rates. Eleven (42.3%) of these papers compared POCT with standard testing and reported significant decreases in antibiotic use in patients receiving an influenza positive POCT diagnosis. Three of the studies also assessed differences between positive and negative POCT results, demonstrating that patients positive for influenza by POCT were significantly less likely to receive antibiotics. Eight other studies (30.8%) reported similar findings. There were six (23.1%) studies that found no significant changes in antibiotic prescription with POCT use. (Egilmezer, 2018) - 789/2031 (38.8%) patients had opportunity for antibiotic avoidance (according to PCR and/or PCT results). (Timbrook, 2015) - Inappropriate antibiotics continued in 45% (160/359), but after molecular respiratory tests introduced there was a decrease in inpatient antibiotics (after viral detection) from 51.3% (81/158) to 39.3% (79/201), p=0.024. In children, this went from 44.5% (53/119) to 28.8% (40/139), p=0.009. (Keske, 2018) |
| Cost | Economic data related to diagnostic testing and/or downstream effects of testing. | May be related to patient care activities (i.e. decision to provide antibiotics, decision to perform additional testing) or to direct laboratory costs. | - Reduction shown in a majority of high quality studies (Vos, 2019) - Median cost of antimicrobial therapy was lower in FilmArray patients vs. standard patients ($3037 vs. $7952, p=0.02). (Gilbert, 2016) - Mean cost (related to reduced oseltamivir usage in flu negative patients) was ~$34.16 per patient. The amount of oseltamivir utilized after we began using the FilmArray RP (24 h a day/7 days per week) would cost US$9564.80 (if all 112 influenza-positive patients received the standard 75 mg every 12 h dose for a duration of 5 days), in addition to US$2527.84 for those that would have received empiric therapy for a duration of 2 days prior to discontinuation following a negative influenza result, totaling US$12,009.64 in expenditure on oseltamivir during this time period. (Pettit, 2015) - Estimated cost savings of using RRP was $178 less per sample. (Rogers, 2015) - $8308/1000 patient-days [SD 10165] vs $11890/1000 [11712] (p=0.02) (Gilbert, 2016) |
| Etiologic Determination (including negative bacterial detection) | Outcomes related to having a pathogen detected or ruled-out. | Discriminating viral pathogens from bacterial/fungal causes of disease may impact patient management decisions and clinical outcomes.  Viral etiologies may often require only supportive care, and often do not require antibiotics or antiviral treatment.  Viral etiologies may require less hospitalization.  Clinical presentation/ suspicion that matches negative bacterial detection, may guide antibiotic treatment decisions. | **Positive Detection Effect** |
|  |  |  | - Post-hoc analysis showed shorter antibiotic course in patients with a positive result (6.2 d, SD 4.8) than in those with a negative result (8d, SD 5.3), p=0.0033. Additionally LOS was shorter with a positive result (4.7d, SD 4.6) than with a negative result (6.5d, SD 7.2), p=0.0085. (Brendish, 2017). - Trend toward higher rates of discharge from the ED for patients testing positive for non-influenza viruses on FilmArray than for the rapid antigen group (44% versus 17%; P 0.067). Significantly more patients who tested positive by FilmArray for noninfluenza viruses were discharged from the ED before arrival on the ward, despite being initially slated for admission (21% versus 5%; P= 0.049) (Rappo, 2016) - Patients who were positive only for RSV in the post-RRP group showed a shorter test turnaround time (P = .001), more patients with a result in the ED (P = .001), a longer ED stay (P=.01), and a decreased duration of antibiotic use by 0.4 day (P = .02). The inpatient LOS was shorter for the group with a positive viral test result following implementation of the RRP (3.5 days pre-RRP versus 3.2 days post-RRP, P =.03). There was no difference in the inpatient LOS for patients with a negative result regardless of whether the test was before or after RRP implementation (P=.88). This may be related to providers being more comfortable discharging patients with a known/confirmed viral pathogen. Patients with a positive viral test result in the post-RRP group were prescribed antibiotics for less time (3.2 days pre-RRP versus 2.7 days post-RRP, P = .001) and were in isolation for a shorter period (82 hours pre-RRP versus 75 hours post-RRP, P = .03) than patients tested before RRP implementation. (Rogers, 2015) - Detection of viruses does not rule-out other disease causes. (Vos, 2019; Brendish, 2017) - Positive viral detection may allow for earlier discontinuation of empiric antibiotics in those with exacerbation of airway disease. (Brendish, 2017). - Positive viral detection removes diagnostic uncertainty, which enhances use of appropriate antiviral prescriptions, which also may lead to eliminating unnecessary antibiotics. (Egilmezer, 2018) |
|  |  |  | **Negative Detection Effect** |
|  |  |  | - Negative results may prevent or shorten the use of isolation facilities and neuraminidase inhibitors. (Brendish, 2017). |
| Infection Control measures | Isolation precautions are required certain pathogens. Infection control measures entail the amount of resources (e.g. time, staff, PPE) needed to effectively isolate infected patients to prevent nosocomial infection. | While there are many facets of infection control measures, studies typically report outcomes related to the time in infection control precautions- interventions may vary significantly from site to site depending on resource availability, however a common denominator is the time spent in isolation, as this has a bearing on all other resources. | - Improved appropriate use of isolation rooms, as evidenced by greater proportion of patients with confirmed influenza in isolation, quicker time to isolation with more rapid confirmation of influenza, and quicker time to de-isolation in those with negative influenza, in those with rapid molecular testing than those with conventional testing. Use of isolation facilities 33% vs 25% (p=0.12) overall and 74% vs 57% (p=0.24) for influenza positive. (Brendish, 2017) - Overall, no change in time in isolation after implementing RRP (73h pre, 70h post, p=0.27). Subgroup analysis of virus positive patients, showed less time in isolation post-RRP (82h pre, 74h post, p=0.03). (Rogers, 2015) |
| Admission avoidance | Ability of test result to prevent an unnecessary hospitalization. | Highly dependent on the inclusions/exclusion criteria of the study population. Multiple studies only include patients that have been admitted to the hospital. While IP outcome measures are important and in need of further study, fewer studies examine diagnostic tests’ ability to avoid becoming and IP. | - Non-significant increase in proportion of ED discharge rates after introduction of FilmArray panel (61% post FilmArray, 50% pre-FilmArray), p=0.25. (Rappo, 2016) - Despite being “initially slated for admission” more patients who tested positive for non-influenza viruses were discharged from the ED after introduction of the FilmArray panel (21% vs. 5%), p=0.049. Difference in discharge rates became more pronounced and significant when comparing the influenza patients tested by FilmArray to the influenza patients with discordant results in season 1 (61% versus 37%; P = 0.036). (Rappo, 2016) - Multivariate log. regression (adjusting for age, immunosuppression, asthma, and emergency severity index) on patients who were influenza positive had a lower odds ratio of being admitted if tested in the ED when using the FilmArray (OR 0.32, 95% CI 0.1 to 0.98, p=0.046). (Rappo, 2016) - The number of hospital admissions was not reduced by rapid molecular testing (Vos, 2019) - No reduction in hospital admissions (92% vs 92%, p=0.94), ICU admission 3% vs 2% (p=0.36) and 30-day readmission 13% vs 16% (p=0.28) (Brendish, 2017) - 30-day readmission 19% vs 20% (p=0.70) (Andrews, 2017) - ICU admission 0% vs 0% (p=1.00) (Rogers, 2014) |
| Pathogen incidence/ distribution |  |  | - Eleven infants had a dual infection (9.2%). RSV and RV were the most frequent viral pathogens; RSV was detected in 58 infants (48.3%) and RV in 27 infants (22.5%) (Van Leeuwen, 2012) - Only single pathogens were detected by routine testing but FilmArray® detected dual infections in - five samples. FilmArray® also detected coronaviruses, not detected using standard tests (Andrews, 2017) - one or more potential pathogens were detected in 90 of 127 (71%) patients. In 40 of 127 (32%) only a potential bacterial pathogen was found; in 25 of 127 (20%) only a potential viral pathogen was detected; and in 24 of 127 (19%) both viral and bacterial pathogens were found (Gilbert, 2016) - Viral PCR panels detected a respiratory virus in 49 of the 127 38.6%) of the patients (Gilbert, 2016) - Among the patients who have viral pathogens in etiology, 211 (28.2%) were adult, and influenza A (52 of 211) (25%), rhinovirus/ enterovirus (49 of 211) (23%), and influenza B (33 of 211) (16%) were the most commonly detected viruses. Among children, rhinovirus/enterovirus (182 of 536) (34%), adenovirus (92 of 536) (17%), influenza A (66 of 536) (12%), RSV (63 of 536) (12%), and influenza B (53 of 536) (10%) were the most commonly detected viruses (Fig. 2). In 71 patients out of 747 (9.5%), multiple viral pathogens were detected (Keske, 2018) - Influenza A and B viruses were the predominant viruses identified in season 1 (158/198 [80%]) and season 2 (54/139 [39%]), followed by RSV (15%) in season 1 and rhinovirus/enterovirus (30%) and metapneumovirus (19%) in season 2 (Rappo, 2016) - The virus detected most commonly was RSV both before and after RRP implementation. However, influenza A was found in 82 positive post-RRP samples (13.7%), while the virus was absent from positive pre-RRP samples. Viruses detected in the post-RRP group that were not tested for in the pre-RRP group include rhinovirus/enterovirus (126 [21.1%]), coronavirus NL62 (7 [1.2%]), and adenovirus (4 [0.7%]). Parainfluenza 1 through 3 were tested for in both groups, with 3 positive samples (1.4%) in the pre-RRP group compared with 21 positive samples (2.7%) in the post-RRP group. Parainfluenza 4 was only tested for in the post-RRP group and was identified in one patient (0.002%). Human metapneumovirus was tested for in less than 1% of samples in the pre-RRP group, and all were negative. After RRP implementation, the number of samples positive for human metapneumovirus was 57 (9.5%) (Rogers, 2015) - The most frequently detected respiratory viruses were influenza A/B (34% of total) and rhinovirus/enterovirus (Timbrook, 2015) |

**Supplementary Table 4.** Description of outcome domains and summary of examples

| Outcome Domains | Description | Examples |
| --- | --- | --- |
| Operational | Test performance | - Turnaround time (TAT) - Sensitivity, specificity |
| Patient | Clinical patient outcome change based on management changes | - De-escalation of antibiotic therapy - Appropriate antiviral use - Number of days with antibiotic treatment - 30-day mortality - Hospital length of stay - 30-day readmission - Antibiotic avoidance |
| Economic | Cost avoidance related to clinical management and/or medical outcomes impact | - Isolation rooms - Lab costs |
| Societal | Aggregate evaluations with public health impacts | - Reduction in resistance (MDROs) for a given time period - Quality-adjusted life years |
